# Treg-derived TGF-β1 dampens cGAS-STING signaling to downregulate the expression of class I MHC complex in multiple myeloma

**DOI:** 10.1038/s41598-024-62298-3

**Published:** 2024-05-21

**Authors:** Disi Zhang, Dong Zhan, Rui Zhang, Yunyan Sun, Ci Duan, Jiapeng Yang, Jia Wei, Xianshi Li, Yanqi Lu, Xun Lai

**Affiliations:** 1 Department of Hematology, Yunnan Cancer Hospital, The Third Affiliated Hospital of Kunming Medical University, Peking University Cancer Hospital Yunnan, No. 519 Kunzhou Road, Xishan District, Kunming, Yunnan Province China; 2 Department of Human Anatomy and Histology and Embrology, School of Basic Medical Sciences, Kunming Medical University, Kunming, Yunnan Province China; 3 Department of Thoracic Surgery, Yunnan Cancer Hospital, The Third Affiliated Hospital of Kunming Medical University, Peking University Cancer Hospital Yunnan, No. 519 Kunzhou Road, Xishan District, Kunming, Yunnan Province China

**Keywords:** Multiple myeloma (MM), Regulatory T cell (Treg), Major histocompatibility complexes (MHC), TGF-β1, cGAS-STING, Cancer microenvironment, Myeloma

## Abstract

Multiple myeloma (MM) progression involves diminished tumor antigen presentation and an immunosuppressive microenvironment, characterized by diminished expression of major histocompatibility complexes (MHC) class I molecule and elevated programmed death ligand 1 (PDL1) in MM cells, along with an enriched population of regulatory T cells (Tregs). To investigate Treg's influence on MM cells, we established a co-culture system using Tregs from MM patients and the MM cell lines (MM.1S and SK-MM-1) in vitro and assessed the effects of intervening in the relevant pathways connecting Tregs and MM cells in vivo. In vitro, Tregs induced transforming growth factor beta-1 (TGF-β1) production, downregulated MHC I members, and increased PDL1 expression in MM cells. Treg-derived TGF-β1 suppressed the cGAS-STING pathway, contributing to the loss of MHC I molecule expression and PDL1 upregulation. Correspondingly, neutralizing TGF-β1 or activating the cGAS-STING pathway restored MHC I and PDL1 expression, effectively countering the pro-tumorigenic effect of Tregs on MM cells in vivo. These data elucidated how Tregs influence tumor antigen presentation and immunosuppressive signal in MM cells, potentially providing therapeutic strategies, such as neutralizing TGF-β1 or activating the cGAS-STING pathway, to address the immune escape and immunosuppressive dynamics in MM.

## Introduction

Multiple myeloma (MM) is the second most common hematologic malignancy, which initiates with monoclonal gammopathy of unknown significance and develops into a spectrum of plasma cell dyscrasias including leukemia and extramedullary myeloma^1,2^. Individuals with unhealthy lifestyles including chronic drinking and smoking, as well as genetic mutations, are predisposed to MM^3,4^. A variety of chemotherapeutic drugs targeting different cellular processes have been approved for MM treatment, including dexamethasone, lenalidomide, doxorubicin, cyclophosphamide, and vincristine. However, the development of drug resistance is a common issue compromising the treatment outcome of all clinical drugs^5,6^.

Similar to solid tumors, a prevalent characteristic of MM is the alteration and functional compromise of the patient's immune system^7^. The immunosuppression state is linked to the changes in the abundance and functionality of principal immune cells^8^. Immunotherapies including immune checkpoint blockade (ICB) and chimeric antigen receptor T (CAR-T) cell therapy have been established to boost anticancer immunity in MM patients^9,10^. Nevertheless, a portion of MM patients are unresponsive to immunotherapy, which may be due to multiple factors including the highly immunosuppressive bone marrow microenvironment and the downregulation of tumor antigen presentation by MM cells^11,12^.

The malignant progression of MM is frequently associated with the dampening of antigen presentation^13,14^. For example, there is evidence that major histocompatibility complexes (MHC) class I chain-related protein A antibodies and shedding are associated with the progression of multiple myeloma, which could undermine the effective recognition of tumor neoantigens^15^. Reduced expression of tumor antigens or MHC together with defective antigen processing and presentation facilitates the immune escape from cytotoxic T lymphocytes (CTLs)^16^. Besides, MM cells could express high levels of immunosuppressive signal molecules such as Programmed Death Ligand 1 (PDL1) to curtail the activation of antitumor immune responses^17,18^. Further, MM patients are characterized by the immunosuppressive bone marrow microenvironment enriched with suppressive dendritic cells (DCs), myeloid-derived suppressor cells (MDSCs), macrophages, and regulatory T cells (Tregs)^19^. Among the immunosuppressive cells, myeloma-driven Treg expansion largely suppressed the activation of CTLs^20^. However, the interplay between Tregs and MM cells remains to be fully elucidated.

In this study, we attempted to investigate how Tregs influence tumor antigen presentation and immunosuppressive signal in MM cells. We established a co-culture system of Tregs isolated from the blood samples of MM patients and MM cell lines, to investigate the mechanism by which Tregs could impact the expression of class I MHC members and PDL1 in MM Cells in vitro. Additionally, we assessed the effects of intervening in the relevant pathways connecting Tregs and MM cells in vivo.

## Results

### Treg expansion is associated with increased IL-10 and TGF-β1 production in MM patients

**Figure 1:**
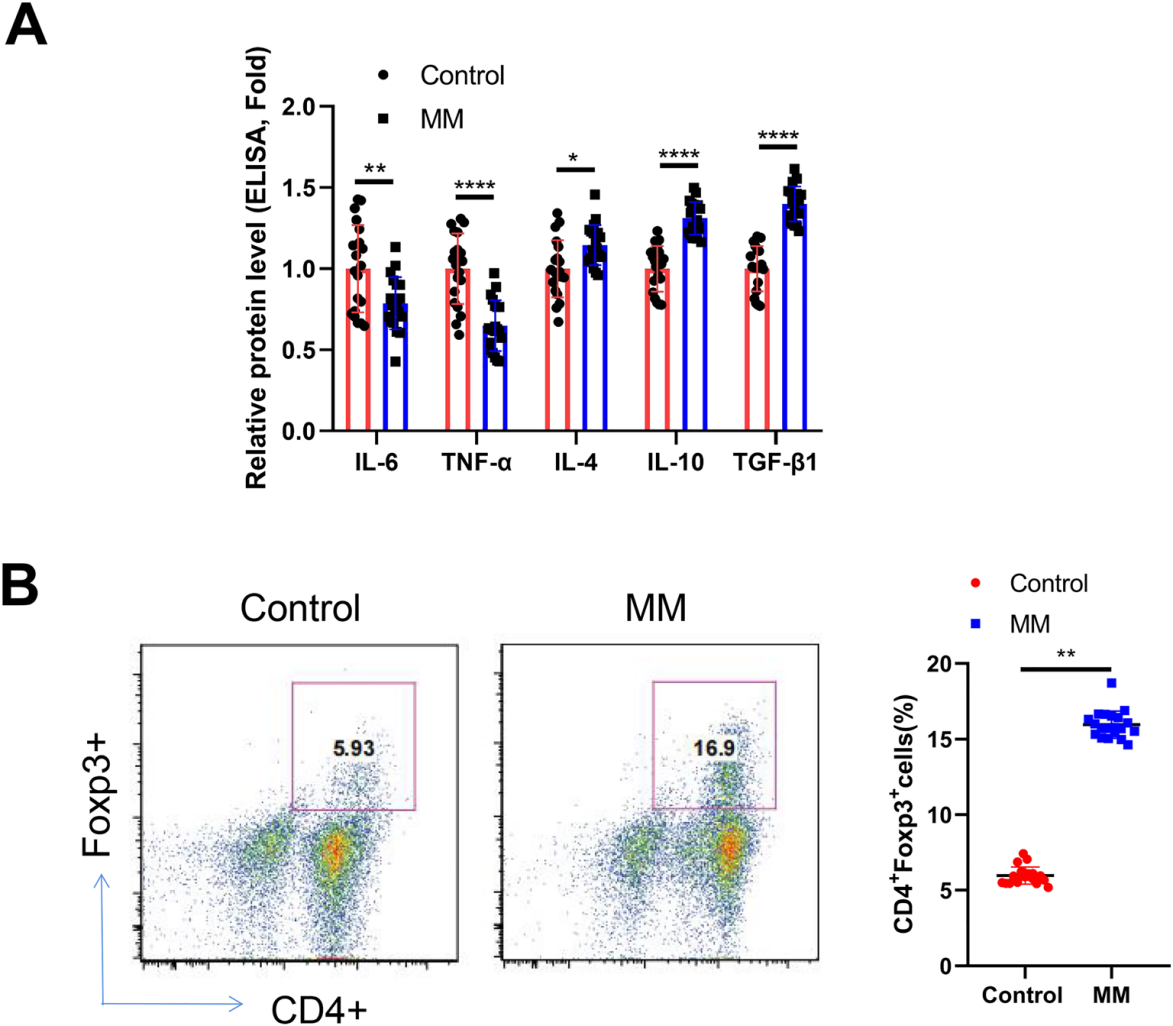
Treg expansion is associated with the elevated production of Il-10 and TGF-β1 in MM patients. (**A**) ELISA analysis of IL-6, TNF-α, IL-4, IL-10, and TGF-β1 in the blood sample of the healthy controls and MM patients. The mean value of each cytokine in the control groups was set as 1, and the values in the MM group were normalized against the mean value of the control group. (**B**) Detection of Tregs using CD4 surface staining and FoxP3 intracellular staining in the blood sample of the healthy controls and MM patients. (n = 20 samples in each group; n = 3 technical replicates for ELISA measurement for each sample; Student’s t test). *P < 0.05, **P < 0.01, ***P < 0.001, ****P < 0.0001.

To compare the cytokine profiles between the healthy controls and MM patients, peripheral blood samples were collected from 20 individuals in each group, and the relative levels of IL-6, TNF-α, IL-4, IL-10, and TGF-β1 were measured. MM patients exhibited a significant decrease in IL-6 and TNF-α levels, while the anti-inflammatory cytokines IL-10 and TGF-β1 showed a marked increase in their blood samples (Fig. 1A). Additionally, the detection of Tregs using CD4 surface staining and FoxP3 intracellular staining revealed a significant increase in Tregs within the CD4^+^ cell population (Fig. 1B). These findings suggest the presence of an immunosuppressive microenvironment in the blood of MM patients.

### Tregs induce downregulation of class I MHC complex and upregulation of PDL1 in MM cells

**Figure 2:**
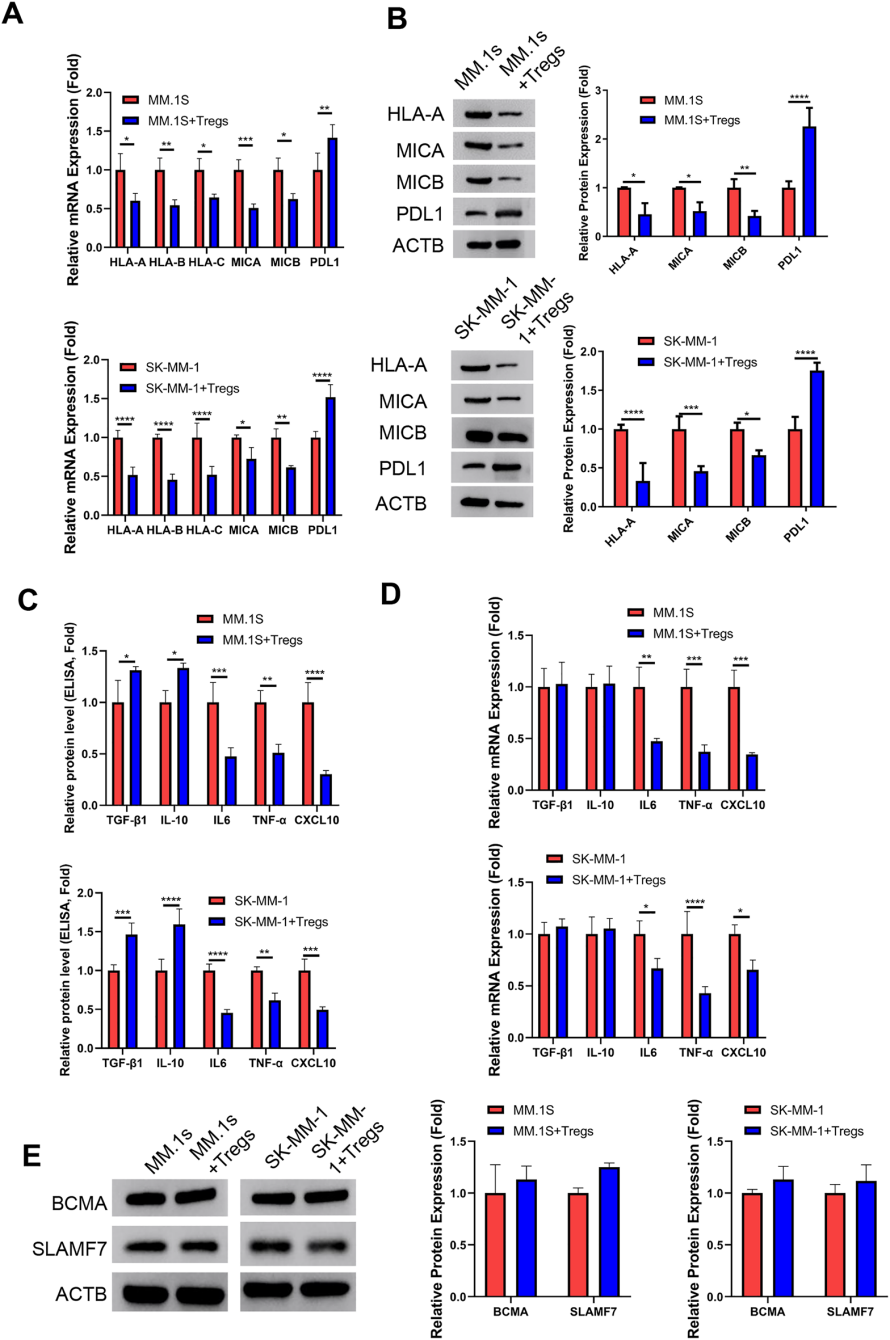
Tregs induce the downregulation of MHC complexes and promote the expression of PDL1 in MM cells. To investigate the impact of Tregs on the antigen presenting molecules in MM cells, human MM cell lines (MM.1S and SK-MM-1) were co-cultured with isolated Tregs from the MM patients at a ratio of 1:0.5 for 24 h. (**A**) qRT-PCR analsyis of HLA-A, HLA-B, HLA-C, MICA, MICB, and PDL1 in MM cells in the absence or the presence of Tregs. (**B**) Western blot analysis of HLA-A, MICA, MICB, and PDL1 in MM cells in the absence or the presence of Tregs. (**C**) ELISA analysis of IL-6, TNF-α and CXCL10 in the cell culture supernatant. (**D**) qRT-PCR analyses of IL-10, TGF-β1, IL-6, TNF-α and CXCL10 mRNAs in MM cells. (**E**) Western blot analysis of the protein levels of SLAMF7 and BCMA in MM cells in the absence or the presence of Tregs. (n = 3 independent experiments; n = 3 technical replicates for ELISA and qRT-PCR measurement in each experimental sample. Student’s t test). *P < 0.05, **P < 0.01, ***P < 0.001, ****P < 0.0001.

To investigate the impact of Tregs on the antigen-presenting molecules in MM cells, we conducted co-cultures using human MM cell lines (MM.1S and SK-MM-1) and isolated Tregs from MM patients for 24 h. Compared to the MM cell culture alone, cells co-cultured with Tregs exhibited a significant downregulation of class I MHC complex members (HLA-A, HLA-B, HLA-C, MICA and MICB), while the expression level of immunosuppressive factor PDL1 was upregulated in the MM cells with Treg co-culture (Fig. 2A,B). As revealed by ELISA analysis, the co-culture with Tregs led to a reduction in the levels of IL-6, TNF-α and CXCL10 in the cell culture supernatant. Conversely, the levels of IL-10 and TGF-β1 were elevated (Fig. 2C). qRT-PCR analyses in MM cells demonstrated no changes in IL-10 and TGF-β1 mRNA levels upon co-culture with Tregs, while the transcription of IL-6, TNF-α, and CXCL10 mRNAs was suppressed in the presence of Tregs (Fig. 2D). We further examined the protein levels of SLAMF7 and BCMA (CAR-T therapy targets in MM cells) in two MM cell lines. We did not find significant changes of these two proteins with or without Treg co-culture (Fig. 2E). Together, these findings suggest that Tregs downregulate class I MHC complex and increases the expression of PDL1 in MM cells.

### Tregs suppress the cGAS-STING pathway in MM cells

**Figure 3:**
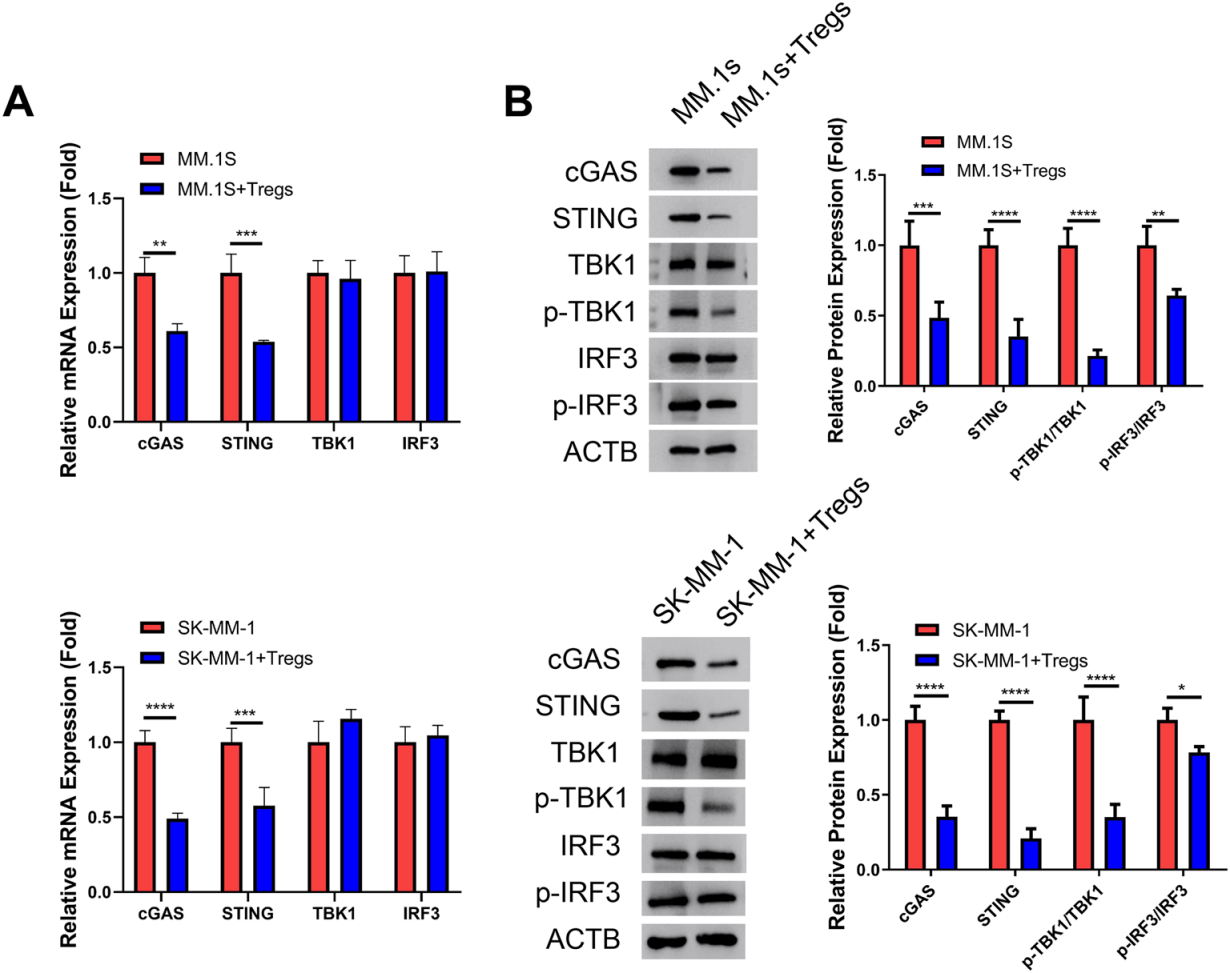
Tregs dampen the cGAS-STING pathway in MM cells. To investigate the impact of Tregs on the antigen presenting molecules in MM cells, human MM cell lines (MM.1S and SK-MM-1) were co-cultured with isolated Tregs from the MM patients at a ratio of 1:0.5 for 24 h. (**A**) qRT-PCR analysis of the mRNA levels of cGAS, STING, TBK1 and IRF3 in human MM cell lines (MM.1S and SK-MM-1) with or without Tregs co-culture. (**B**) Western blot analysis of the protein levels of cGAS, STING, TBK1, p-TBK1, IRF3 and p-IRF3 in the MM cells with or without Tregs co-culture. (n = 3 independent experiments; n = 3 technical replicates for qRT-PCR measurement in each experimental sample. Student’s t test). *P < 0.05, **P < 0.01, ***P < 0.001, ****P < 0.0001.

Given that the cGAS-STING signaling pathway drives the expression of IL-6, TNF-α and CXCL10, we next attempted to investigate the impact of Tregs on the cGAS-STING pathway in MM cells. qRT-PCR analysis revealed a decrease in the mRNA levels of cGAS and STING in both MM.1S and SK-MM-1 cells upon co-culture with Tregs, while the mRNA levels of TBK1 and IRF3 remained unchanged (Fig. 3A). We further examined the activation state of cGAS-STING signaling by detecting the phosphorylation levels of TBK1 and IRF3 in MM cells in the presence or absence of Tregs. With the decreased protein levels of cGAS and STING in the presence of Tregs, the phosphorylation levels of TBK1 and IRF3 were also diminished in MM cells in the presence of Tregs (Fig. 3B). Thus, this could implicate that Tregs dampen cGAS-STING pathway in MM cells.

### STING agonist counteracts Treg-mediated effects on MM cells

**Figure 4:**
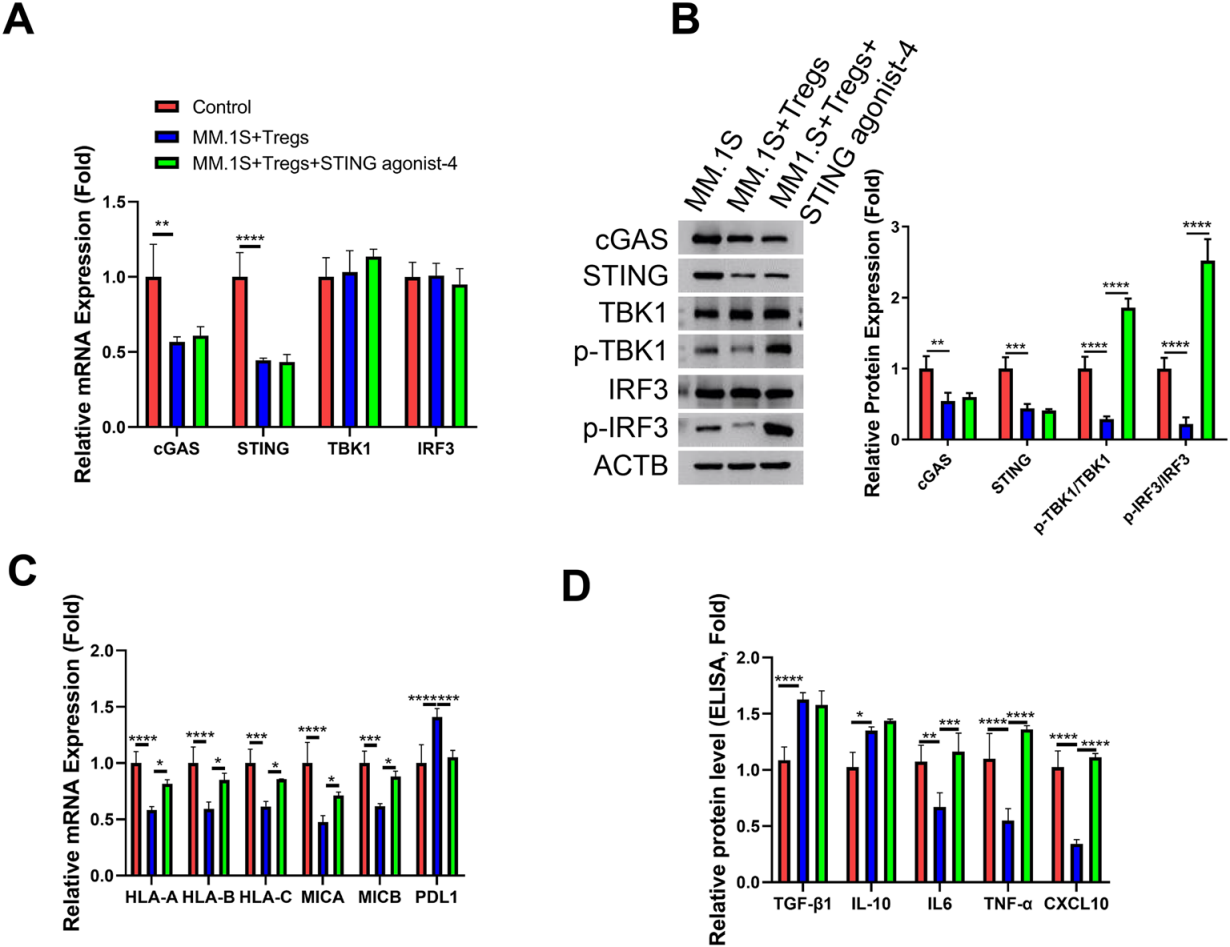
STING agonist counteracts the effect of Tregs on MM cells. STING agonist-4 was applied at 1 μM in the MM cells in the presence of Tregs co-culture (MM cells were co-cultured with isolated Tregs at a ratio of 1:0.5 for 24 h). (**A**) qRT-PCR analysis of cGAS, STING, TBK1 and IRF3 at the mRNA levels. (**B**). Western blot analysis of the protein levels of cGAS, STING, TBK1, p-TBK1, IRF3 and p-IRF3. (**C**). qRT-PCR analsyis of HLA-A, HLA-B, HLA-C, MICA, MICB, and PDL1. (**D**). ELISA analysis of IL-10, TGF-β1, IL-6, TNF-α and CXCL10 in cell culture supernatant. (n = 3 independent experiments; n = 3 technical replicates for ELISA and qRT-PCR measurement in each experimental sample. One-way ANOVA). *P < 0.05, **P < 0.01, ***P < 0.001, ****P < 0.0001.

Next, we investigated whether the reactivation of cGAS-STING signaling could counteract the impact of Tregs on MM cells. STING agonist-4, a stimulator of STING receptor that has been demonstrated to induce the phosphorylation of IRF3 and STING^21^, was administered to MM cells in the presence of Tregs. STING agonist-4 did not influence the mRNA levels of cGAS, STING, TBK1 and IRF3 (Fig. 4A). Nevertheless, its application successfully reactivated cGAS-STING signaling in MM cells co-cultured with Tregs, as evidenced by the restoration of TBK1 and IRF3 phosphorylation (Fig. 4B). Additionally, STING agonist-4 was capable of reinstating the expression of class I MHC complex members while reducing the expression of PDL1 in MM cells (Fig. 4C). ELISA analysis further revealed that although the levels of IL-10 and TGF-β1 remained unaffected by STING agonist-4 (as these cytokines are produced by Tregs), STING agonist-4 treatment significantly increased the production of IL-6, TNF-α and CXCL10 in MM cells when co-cultured with Tregs (Fig. 4D). These data indicate that the Treg-mediated suppression of cGAS-STING signaling contributes to the downregulation of MHC complex members and the upregulation of PDL1 in MM cells.

### TGF-β1 neutralization curtails the impact of Tregs on MM cells

**Figure 5:**
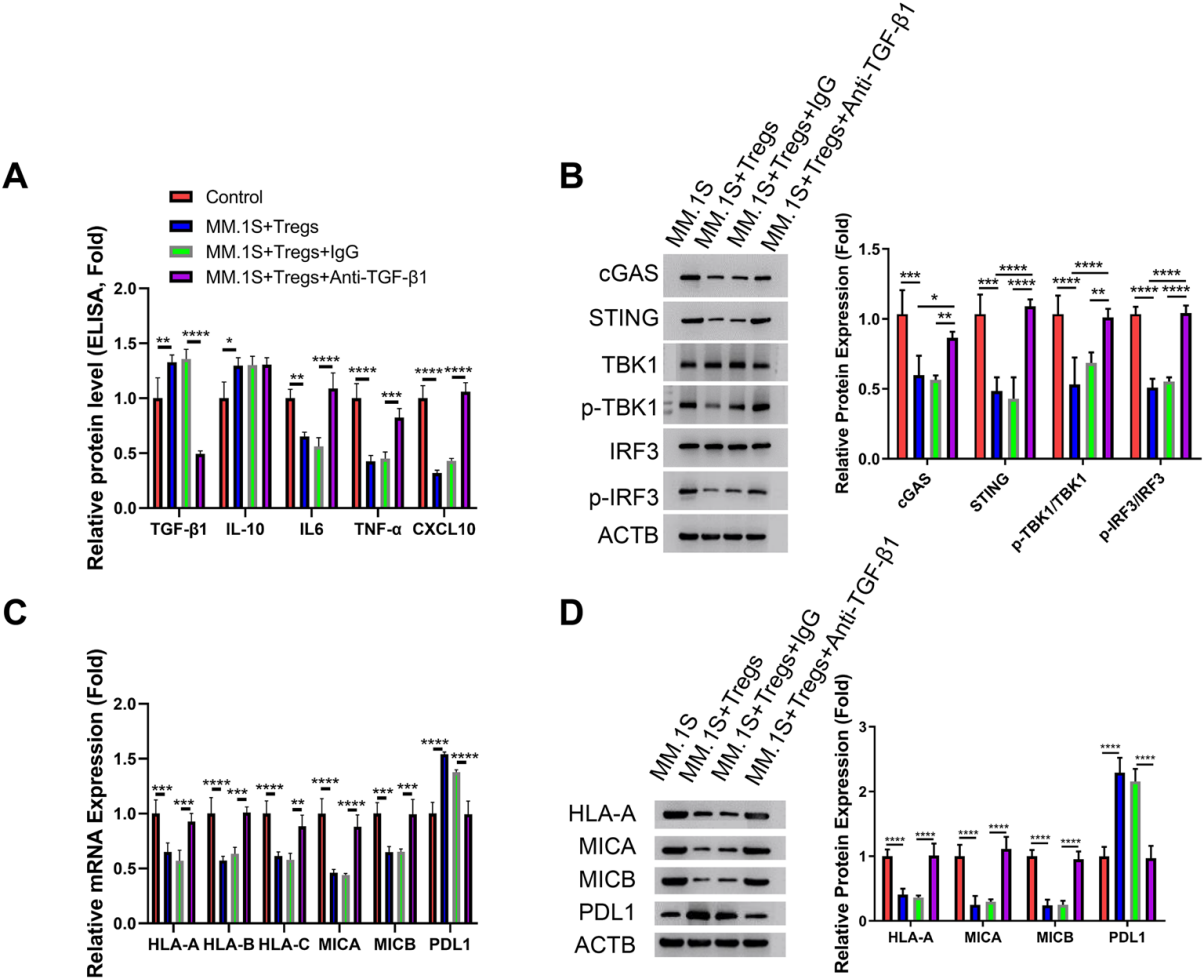
TGF-β1 neutralization mitigates the effect of Tregs on MM cells. TGF-β1 neutralizing antibody was applied at 1 μg/mL in the co-culture system of MM cells and Tregs, with the IgG isotype as the control (MM cells were co-cultured with isolated Tregs at a ratio of 1:0.5 for 24 h). (**A**). ELISA analysis of IL-10, TGF-β1, IL-6, TNF-α and CXCL10 in cell culture supernatant. (**B**). Western blot analysis of the protein levels of cGAS, STING, TBK1, p-TBK1, IRF3 and p-IRF3. (**C**). qRT-PCR analsyis of HLA-A, HLA-B, HLA-C, MICA, MICB, and PDL1. (**D**). Western blot analysis of HLA-A, MICA, MICB, and PDL1. (n = 3 independent experiments; n = 3 technical replicates for ELISA and qRT-PCR measurement in each experimental sample. One-way ANOVA). *P < 0.05, **P < 0.01, ***P < 0.001, ****P < 0.0001.

We hypothesized that Treg-derived TGF-β1 may account for the suppression of cGAS-STING signaling in MM cells. To test this hypothesis, we applied a TGF-β1 neutralizing antibody in the co-culture system, with the IgG isotype serving as the control. TGF-β1 neutralizing antibody effectively decreased the levels of labile TGF-β1 in the supernatant, while IL-10 level remained unchanged (Fig. 5A). Interestingly, TGF-β1 neutralization promoted the expression of cGAS-STING signaling-dependent cytokines (IL-6, TNF-α, and CXCL10) in MM cells co-cultured with Tregs. Western blot analysis further confirmed that TGF-β1 neutralization increased the expression of cGAS and STING in MM cells and reactivated the phosphorylation of TBK1 and IRF3 (Fig. 5B). Besides, neutralizing TGF-β1 restored the expression of class I MHC complex members and reduced the expression of PDL1 in MM cells co-cultured with Tregs (Fig. 5C,D). These findings point that Treg-derived TGF-β1 induces the suppression of cGAS-STING signaling in MM cells.

### The pro-tumorigenic effect of Tregs is suppressed by STING agonist and TGF-β1 neutralization

**Figure 6:**
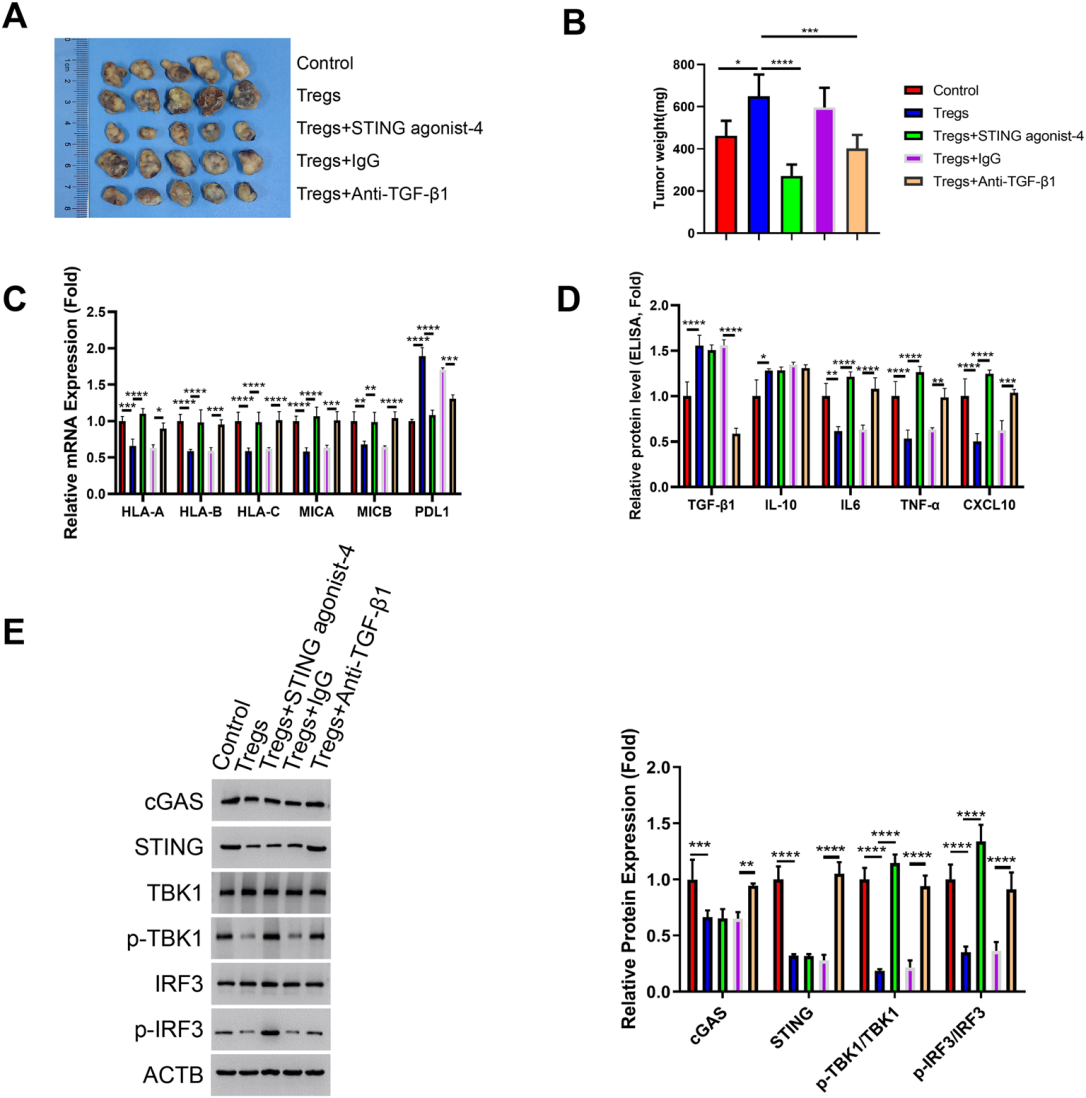
STING agonist and TGF-β1 neutralization counteract the pro-tumorigenic effect of Tregs. The nude mice were injected with MM.1S cells, MM.1S cells plus Tregs, MM.1S cells plus Tregs and STING agonist-4, MM.1S cells plus Tregs and IgG isotype or MM.1S cells plus Tregs and TGF-β1 neutralizing antibody (n = 5 in each group). The tumor samples were harvested from nude mice on day 35. (**A**). Xenograft tumor images in each experimental group. (**B**). The summary of tumor weight in each group. (**C**). qRT-PCR analsyis of HLA-A, HLA-B, HLA-C, MICA, MICB, and PDL1 in the tumor tissues. (**D**). ELISA analysis of IL-10, TGF-β1, IL-6, TNF-α and CXCL10 in the tumor tissues. (**E**). Western blot analysis of the protein levels of cGAS, STING, TBK1, p-TBK1, IRF3 and p-IRF3 in the tumor tissues. (n = 5 animals in each group; n = 3 technical replicates for ELISA and qRT-PCR measurement in each experimental sample. One-way ANOVA).*P < 0.05, **P < 0.01, ***P < 0.001, ****P < 0.0001.

To further elucidate the impact of Treg-mediated cGAS-STING suppression on MM cell tumorigenesis, the nude mice were injected with MM.1S cells, MM.1S cells with Tregs, MM.1S cells with Tregs and STING agonist-4, MM.1S cells with Tregs and IgG isotype or MM.1S cells with Tregs and TGF-β1 neutralizing antibody. The administration of Tregs promoted the tumor formation of MM cells in the nude mice (Fig. 6A,B). Notably, both STING agonist-4 and TGF-β1 neutralizing antibody suppressed the pro-tumorigenic effect of Tregs. In the tumor samples, the administration of STING agonist-4 and TGF-β1 neutralizing antibody also counteracted the effect of Tregs on class I MHC molecule and PDL1 expression of MM cells (Fig. 6C). ELISA analysis also showed STING agonist-4 and TGF-β1 neutralization promoted the expression of cGAS-STING signaling dependent cytokines (IL-6, TNF-α and CXCL10) in MM tumors (Fig. 6D). In addition, TGF-β1 neutralization increased cGAS and STING expression and enhanced TBK1 and IRF3 phosphorylation in the group injected with Tregs. STING agonist-4 exhibited a similar effect on the activation of TBK1 and IRF3 phosphorylation (Fig. 6E). These data suggest that TGF-β1-dependent suppression of cGAS-STING signaling in MM cells contributes to the pro-tumorigenic effect of Tregs. Of note, the activation of cGAS-STING pathway may exert a direct anti-tumorigenic effect on tumor, a potential effect that needs to be clarified by the future study.

## Discussion

In this study, we demonstrated that Treg expansion was associated with elevated production of IL-10 and TGF-β1 in MM patients. The co-culture of Tregs with MM cells resulted in increased TGF-β production, downregulation of class I MHC members, and promoted the expression of PDL1. TGF-β1 derived from Tregs dampened the cGAS-STING signaling pathway in MM cells, contributing to the decreased MHC expression and elevated PDL1 levels in MM cells. Neutralizing TGF-β1 or activating cGAS-STING pathway not only antagonized the effect of Tregs on the tumor antigen presentation and immunosuppressive signal in MM cells, but also mitigated the pro-tumorigenic effect of Tregs in the mouse model.

Tregs are a specialized population of T cells that functions to dampen immune responses and maintain immune homeostasis and self-tolerance^22^. Tregs can suppress T cell proliferation, activation, and inflammatory cytokine production^23^. Thus, Tregs have great therapeutic potential in treating autoimmune and chronic inflammatory diseases^24^. However, in tumor tissues Treg recruitment and expansion created an immunosuppressive hurdle for antitumor immunity^25^. Target intratumoral Tregs becomes an attractive strategy to re-activate antitumor immunity in the tumor microenvironment^26^. In MM patients, myeloma drives the expansion of Tregs in the bone marrow through secreting cytokines^20^. Further, it has been widely reported that Treg expansion is a immunological feature in the peripheral blood of MM patients^20,27,28^. Increased Treg frequency in the blood has also been recognized as an adverse clinical feature that predicts progression in MM patients^29^. Consistent with the observation, we also showed that there is an increased percentage of CD4^+^FoxP3^+^ Tregs in the CD4^+^ T cell population in the blood samples of MM patients. Treg expansion is also associated with the increased levels of anti-inflammatory cytokines (IL-10 and TGF-β1). Neutralizing TGF-β1 not only counteracted the effect of Tregs on MHC complex expression in MM cells, but also suppressed the pro-tumorigenic effect of Tregs in the mouse model. Since peripheral Tregs can be recruited into the tumor microenvironment and the elevated production of immunosuppressive cytokines may also affect the immunity in the tumor tissues^20,25,26^, we envision that Treg-derived cytokines might act systemically both in the blood and bone marrow of MM patients. Future work is required to clarify whether Treg-derived IL-10 exerts a similar effect on MM cells through the same or a different mechanism.

The loss or dampening of antigenicity in MM cells are widely reported^30,31^. This can happen through the downregulation of tumor-specific antigen, antigen shedding, genetic alteration, and the lowered expression of antigen presentation machinery^14,32^. Apart from the suppression of T cell activation and cytokine production, Tregs are also reported to modulate antigen presentation and the co-stimulatory signals on the antigen presenting cells (APCs). For examples, lymphocyte activation gene 3 (LAG3) expressed by Tregs can downregulate the expression of MHC II molecules in the dendritic cells (DCs)^33^. Treg also suppresses B7-2 co-stimulatory molecule and induces the expression of negative regulatory molecules B7-H3 and B7-H4 on DCs^34^. In our study, we showed that Tregs could dampen the expression of MHC I molecules on MM cells in a TGF-β1 dependent manner. The downregulation of MHC I molecule is a well-established cancer immune evasion mechanism^16^. The high level expression of MHC I molecule MICA on bone marrow myeloma cells renders them highly susceptible to the lysis by natural killer cells^35^. Inducible downregulation of MHC class I can lead to natural killer cell tolerance^36^. Thus, Treg induced MHC I molecule downregulation may serve as an immune evasion mechanism in MM cells.

We further showed that Treg-derived TGF-β1 accounts for the downregulation of MHC I molecules in MM cells, since TGF-β1 neutralizing antibody could abrogate the effect of Tregs in the co-culture system. Accumulating evidence suggests that TGF-β signaling plays a pivotal role in regulating MHC molecules in different scenarios. The downregulation of class I MHC molecules by TGF-β1 was demonstrated in fibroblasts isolated from TGF-β1 null mice in an early study^37^. TGF-β1 can also suppress IFN-gamma dependent induction of class II MHC genes by attenuating mRNA transcription^38^. In human and horse mesenchymal stem cells, TGF-β signaling activation dampens antigen processing/presentation genes and MHC I molecule expression in a Smad3-dependent mechanism^39^. Therefore, our findings are in consistence with notion that TGF-β signaling negatively impacts on MHC molecule expression.

cGAS-STING signaling serves as a cytosolic double-stranded DNA (dsDNA) sensor and mounts antiviral innate immunity through activating downstream TBK1/IRF3^40^. Recently, cGAS-STING pathway has become a hot spot in cancer immunotherapy, since STING agonists can synergize with other cancer immunotherapies, including cancer vaccines, immune checkpoint inhibitors and adoptive T cell transfer, to boost the anticancer immunity in advanced cancer treatment^41,42^. However, the interplay of cGAS-STING pathway with other signaling processes and its role in regulating antigen presentation are elusive. Our data demonstrated that Treg-derived TGF-β1 can transcriptionally suppress the mRNA levels of cGAS and STING in MM cells. Neutralizing TGF-β1 and the administration of STING agonist were able to counteract the effect of Tregs on MHC I molecule downregulation in MM cells, as well as suppressing the pro-tumorigenic effect of Tregs on MM cells in mouse model. In agreement with our finding, the activation of cGAS-STING pathway promotes cytotoxic T cell-mediated cancer cell killing by increasing MHC I molecule expression on the surface of cancer cells^43,44^. Together, our findings imply that TGF-β1-cGAS/STING axis is an important regulator of MHC I expression in MM cells. The activation of cGAS/STING pathway is essential for the antigenicity in MM cells. Nonetheless, over-activation of cGAS/STING pathway may cause the hyper-activity in other immune cells and lead to side effect such as cytokine storm^45^. The strategy of selectively activating cGAS/STING pathway in MM cells may solve this concern. Moreover, future work is required to further dissect the mechanisms by which TGF-β1 regulates cGAS/STING expression and how cGAS/STING signaling modulates the expression of MHC I molecules in MM cells.

To conclude, we demonstrated that Tregs could induce the downregulation of class I MHC molecules and promoted the expression of PDL1 in MM cells in a TGF-β1 dependent manner. The suppression of cGAS-STING signaling pathway in MM cells underlies the effect of Treg-derived TGF-β1. Our findings suggest that neutralizing TGF-β1 or activating the cGAS-STING pathway could restore the expression of the antigen presentation system and immunosuppressive signal in MM cells affected by Tregs, potentially providing therapeutic strategies for MM treatment.

## Methods

### Clinical samples

The peripheral blood samples were collected from 20 healthy controls and 20 MM patients at the Department of Hematology of Yunnan Cancer Hospital. All the included subjects were primarily diagnosed with MM, without any clinical treatment. Patients diagnosed with other types of malignancies or had a clinical record of chronic diseases were also excluded from the study. The blood samples were snap-frozen in liquid nitrogen and stored in − 80 °C for further analyses. The collection and use of clinical blood specimens in this study were conducted following the Declaration of Helsinki. The research gained the approval by the Institutional Review Board of Yunnan Cancer Hospital (Kunming, China). All the recruited subjected signed the informed consent.

### Treg isolation and analysis

For Treg isolation from the peripheral blood samples, CD4^+^CD25^+^ Regulatory T Cell Isolation Kit, human (Miltenyi Biotec, CA, USA) was used based on the supplier’s instructions. The isolated Tregs were expanded using Human Treg Expansion Kit (Miltenyi Biotec, CA, USA). For cell surface antibody staining, cells were washed in PBS supplemented with 2% FBS and incubated with fluorophore conjugated antibodies (PB-αCD4, 1:400; Fc-Block (αCD16/32), 1:500) at 4 °C for 10 min. For intracellular staining of FoxP3, cells were then fixed and permeabilized using Foxp3 intracellular staining kit (eBioscience, CA, USA) before being stained with corresponding FoxP3 antibodies (APC-αFoxp3 1:100) for an hour. After three washes in permeabilizing buffer the stained cells were re-suspended in PBS and analyzed on an LSRII flow cytometer (BD Biosciences, CA, USA) (Supplementary figures).

### Cell culture

Human MM cell lines (MM.1S and SK-MM-1) were purchased from Cobioer Biosciences (Nanjing, China). The cells were maintained in McCoy’s medium (Thermo Fisher Scientific, CA, USA) containing 10% FBS (Thermo Fisher Scientific, CA, USA) and 1% penicillin/streptomycin (Hyclone, CA, USA) under the condition of 37 °C and 5% CO_2_. For the co-culture experiment of Tregs and MM cells, MM cells were plated with Tregs at a ratio of 1:0.5 in a 24-well plate for 24 h. For STING stimulation, STING agonist-4 (MedChemExpress, Shanghai, China) was applied at 1 μM. To neutralize TGF-β1, NeutraKine® TGF beta 1 Monoclonal antibody (ProteinTech, IL, USA) was applied at 1 μg/ml, with 1 μg/ml IgG isotype as the control. For molecular characterization of MM cells, Tregs were removed by CD25 magnetic beads (Dynabeads™ CD25, Thermo Fisher Scientific, CA, USA).

### Enzyme-linked immunosorbent assay (ELISA)

The relative levels of IL-6, IL-4, IL-10, TGF-β1, TNF-α, and CXCL10 in the blood sample or cell culture supernatant were determined using corresponding ELISA kits (Sigma, St. Louis, MO, USA). Briefly, the micro-assay plates were coated with corresponding antibodies at 4 °C overnight. 100 μl of samples or standards were added into the capture-antibody-coated plate for 2-h incubation at ambient temperature. After a washing step to remove unbound material, 100 μl of the biotin-labeled detection antibody was added for 1-h incubation, which was followed by the incubation with 50 μl of streptavidin-HRP. Next, 100 μl of chemiluminescent detection reagents were added for signal development and the optical densities of each sample and the standards were measured at 450 nm using a microplate reader (Infinite 200 PRO; Tecan).

### qRT-PCR analysis

The total RNA samples from the plasma and cell specimens were extracted using TRIzol reagent (Qiagen, Shanghai, China), and reverse transcription was performed using the PrimeScript™ RT Reagent Kit (Takara Biotechnology, Otsu, Japan). The resulted cDNA was diluted and analyzed in a 7500 Real Time PCR System (Applied Biosystems, CA, USA) using SYBR premix EX TAQ II kit (Takara Biotechnology, Otsu, Japan). All PCR primers were purchased from Sangon Biotechnology (Shanghai, China). The 2^−ΔΔ^CT method was used to quantify the relative gene expression, with β-actin as the reference gene.

### Western blot

Total protein samples were extracted using the RIPA lysis buffer (Beyotime, Beijing, China) mixed with protease inhibitor and PMSF (ThermoFisher Scientific, CA, USA). The supernatant was then collected after 15 min incubation by centrifugation. After denaturation, the protein samples were subjected to separation in 12% sodium dodecyl sulfate–polyacrylamide gel electrophoresis (SDS-PAGE) and then transferred onto a PVDF membrane (0.22 µm or 0.45 µm, Merck-Millipore, Darmstadt, Germany). 5% skimmed milk (w/v) in the TBST buffer was used to block the membrane for 1 h. The membrane was then incubated with primary antibodies (Abcam, Cambridge, UK): anti-HLA-A (ab52922, 1:1000), anti-beta actin antibody (ab8227, 1:1000), anti-MICA (ab62540, 1:1000), anti-MICB (ab167488, 1:1000), anti-PDL1 (ab205921, 1:1000), anti-cGAS (ab224144, 1:1000), anti-STING (ab181125, 1:1000), anti-TBK1 (ab40676), anti-pTBK1 (ab206124, 1:1000), anti-IRF3 (ab68481, 1:1000), and anti-pIRF3 (ab76439, 1:1000) at 4°C overnight. Afterward, the PVDF membrane was washed with 1× TBST buffer and further labeled with horseradish peroxidase (HRP)-conjugated secondary antibody at room temperature for 1 h (Abcam, ab205718, 1:2000). After 3 washes, the protein bands were developed using the ECL chemiluminescent solution (Beyotime, Beijing, China). The intensities of target protein bands were normalized to that of beta-actin (loading control).

### Animal model

Balb/c nude mice (male, 4–5 weeks old) were purchased from the Shanghai Laboratory Animal Center (SLAC) Co., Ltd. (Shanghai, China) and housed at the SPF facility. All animal procedures were performed according to the guidelines approved by the Institutional Animal Care and Use Committee (IACUC) of Yunnan Cancer Hospital (Kunming, China). The following experimental groups were established: control (injected with 1 × 10^6^ MM.1S cells); Tregs group (injected with 1 × 10^6^ MM.1S cells and 5 × 10^5^ Tregs); Tregs + STING agonist-4 group (injected with 1 × 10^6^ MM.1S cells and 5 × 10^5^ Tregs, and administrated with STING agonist-4 at 5 mg/kg every week). Tregs + IgG group (injected with 1 × 10^6^ MM.1S cells and 5 × 10^5^ Tregs, and administrated with IgG isotype at 1 mg/kg every week); Tregs + Anti-TGF-β1 group (injected with 1 × 10^6^ MM.1S cells and 5 × 10^5^ Tregs, and administrated with TGF-β1 neutralizing antibody at 1 mg/kg every week). All the mice were also injected with 1 × 10^6^ of human CD45^+^ lymphocytes. Cells were injected subcutaneously at the right flank of the mice, and STING agonist-4, Anti-TGF-β1 antibody and IgG were administered intraperitoneally. The tumor volume was measured every week. The mice were euthanized by intraperitoneal injection of excessive pentobarbital sodium (200 mg/kg) on day 35 and the tumor samples were collected for further analysis.

### Statistical analysis

Data were analyzed by GraphPad Prism software (GraphPad Software, NY. USA) and expressed as mean ± standard deviation (SD). The analysis between different groups was based on the Student's t-test or one-way ANOVA at a defined statistical threshold of *P* < 0.05.

### Ethic statement

The study was conducted in accordance with the Declaration of Helsinki, and approved by the Institutional Review Board of Yunnan Cancer Hospital (Kunming, China) (protocol code YJZ201904). Written informed consent was obtained from each participant prior to the collection of clinical specimens. The animal experiments were also approved by the Institutional Animal Care and Use Committee (IACUC) of Yunnan Cancer Hospital (Kunming, China) (protocol code kmmu2021692), and were conducted according to the relevant guidelines and regulations. The study was carried out in compliance with the ARRIVE guidelines.

### Supplementary Information

Supplementary Figures.

## Data Availability

The data supporting this study's findings are available from the corresponding author upon reasonable request.
