# Supplementary material for: Treg-derived TGF-β1 dampens cGAS-STING signaling to downregulate the expression of class I MHC complex in multiple myeloma
Source: Sci Rep. 2024 May 21;14:11593. doi: 10.1038/s41598-024-62298-3 (PMC11109281; doi:10.1038/s41598-024-62298-3)
Supplement: Supplementary file 1 — Supplementary Figures. [file 41598_2024_62298_MOESM1_ESM.pdf]

Figure 2

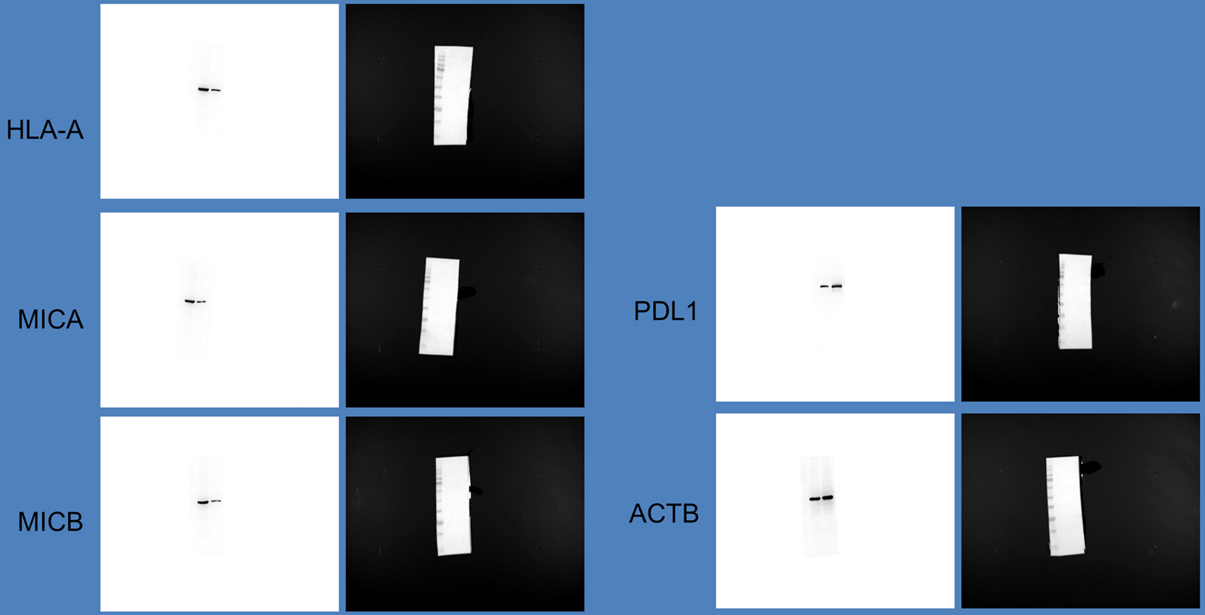

Note: Protein bands are grouped in order from left to right: MM, MM+Tregs

Figure 3

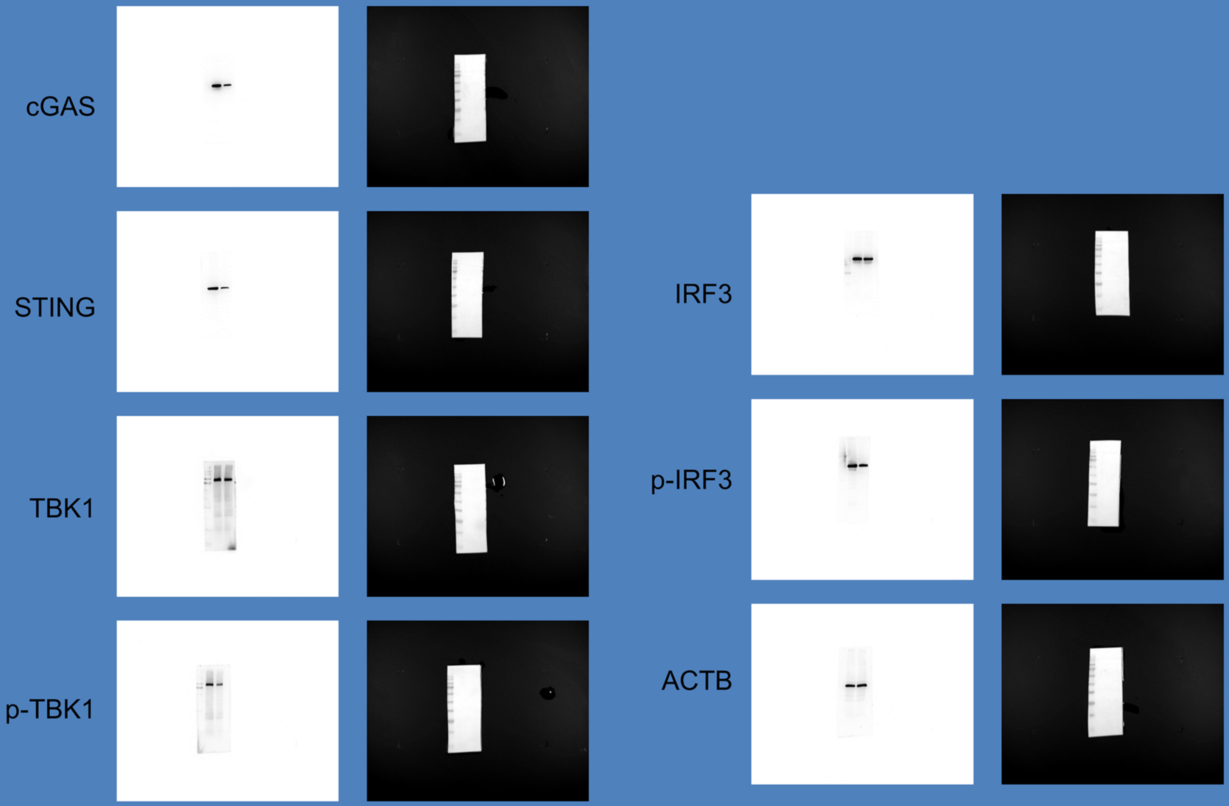

Note: Protein bands are grouped in order from left to right: MM, MM+Tregs

Figure 4

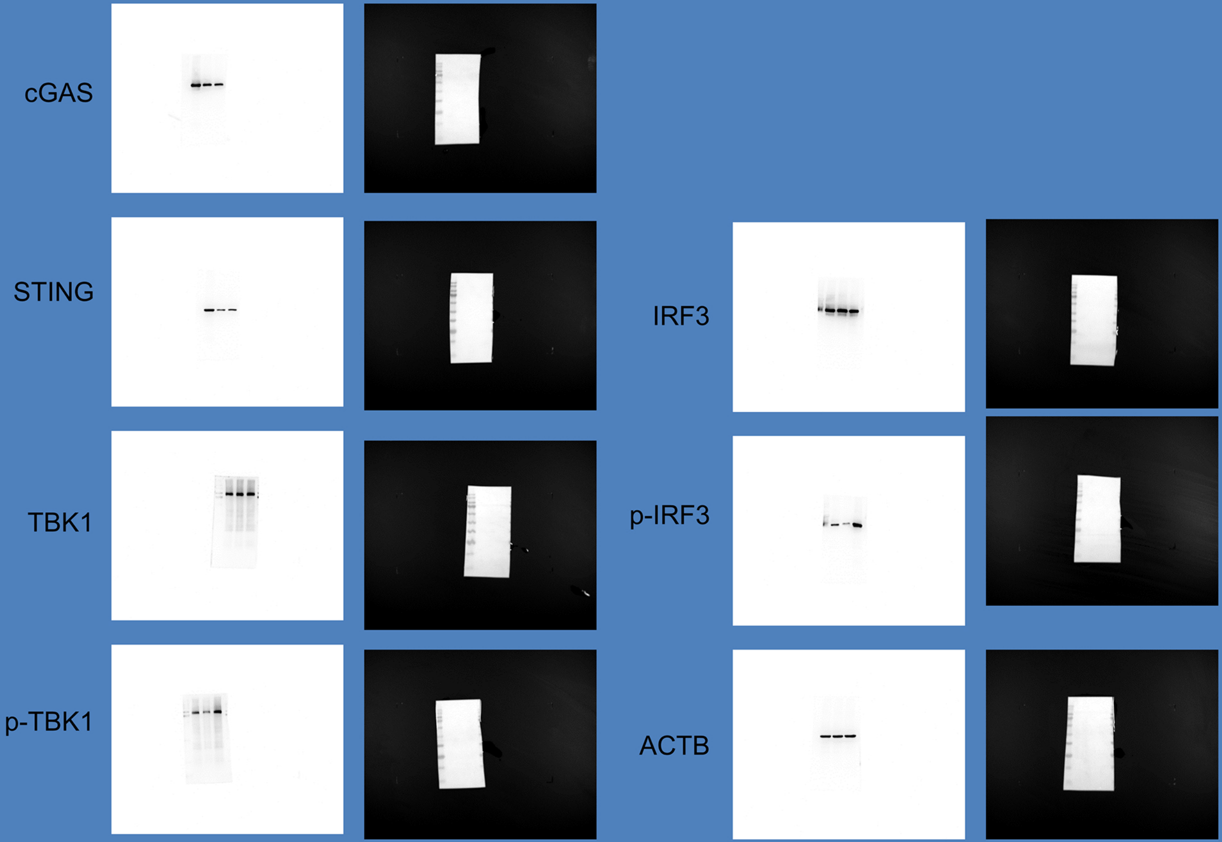

Note: Protein bands are grouped in order from left to right: MM, MM+Tregs, MM+Tregs+STING agonist-4

Figure 5

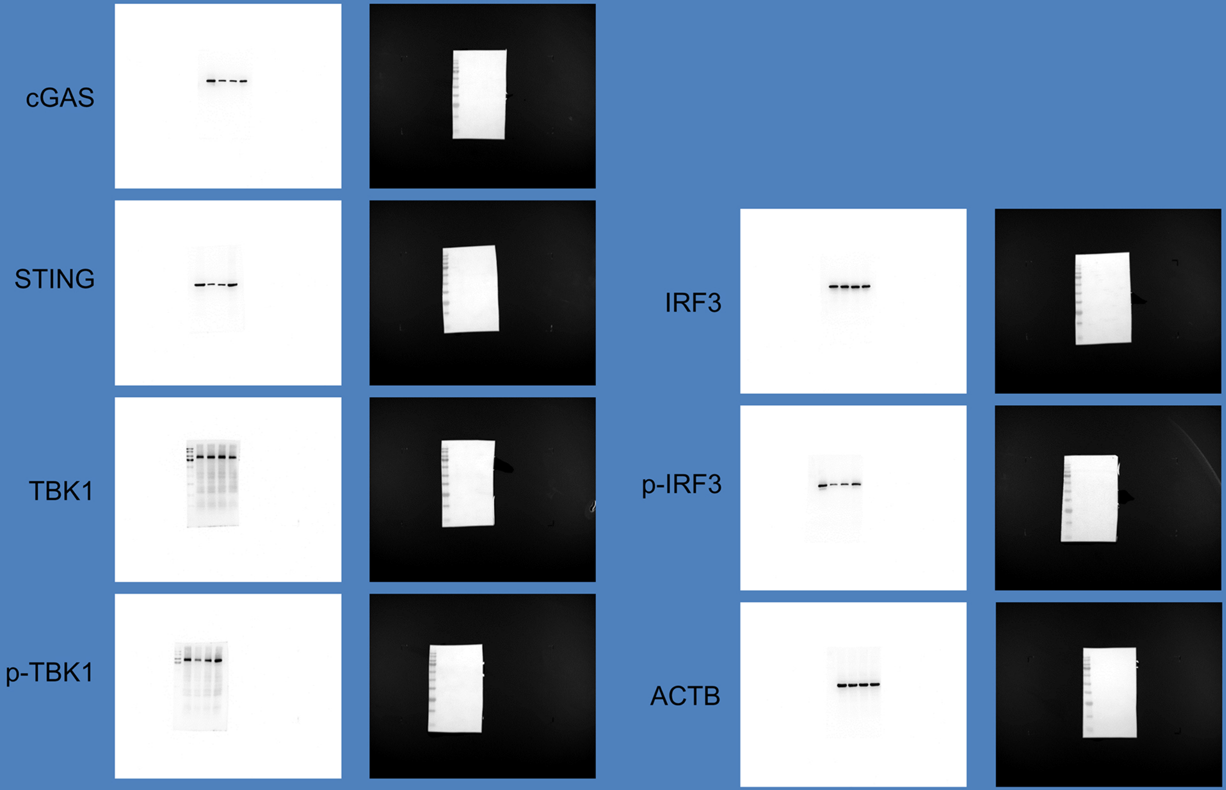

Note: Protein bands are grouped in order from left to right: MM, MM+Tregs, MM+Tregs+IgG, MM+Tregs+Anti-TGF-β1

Figure 5

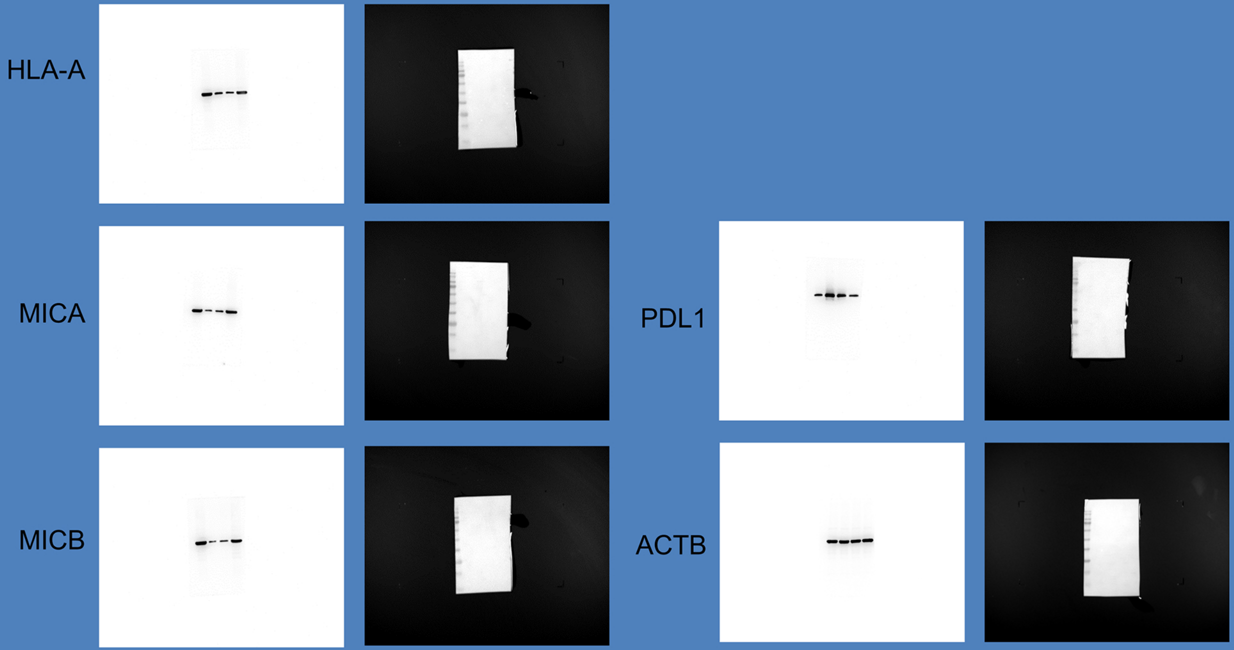

Note: Protein bands are grouped in order from left to right: MM, MM+Tregs, MM+Tregs+IgG, MM+Tregs+Anti-TGF-β1

Figure 6

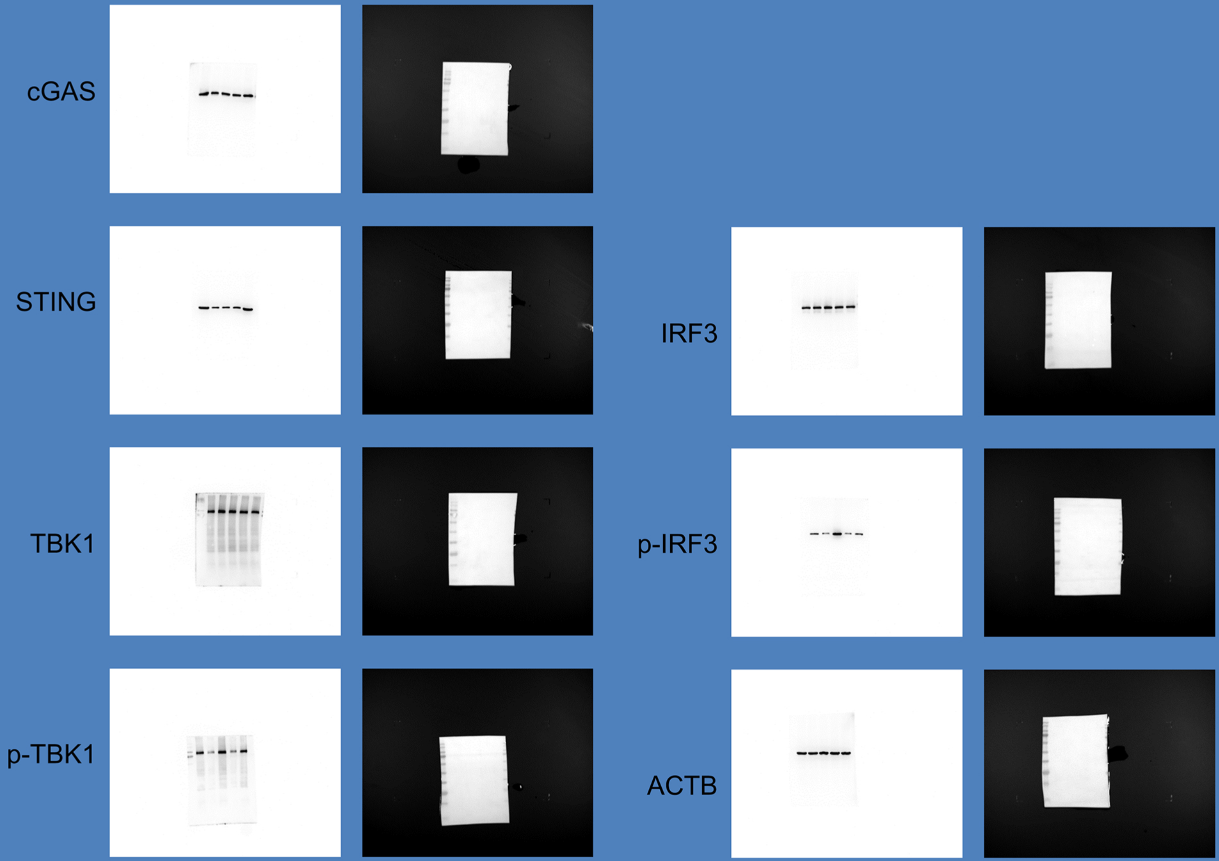

Note: Protein bands are grouped in order from left to right: Control, Tregs, Tregs+STING agonist-4, Tregs+IgG, Tregs+Anti-TGF-β1
